# How to implement routine electronic patient‐reported outcome monitoring in oncology rehabilitation

**DOI:** 10.1111/ijcp.13694

**Published:** 2020-10-02

**Authors:** Lisa M. Wintner, Monika Sztankay, David Riedl, Gerhard Rumpold, Alain Nickels, Thomas Licht, Bernhard Holzner

**Affiliations:** ^1^ Department of Psychiatry, Psychotherapy and Psychosomatics University Hospital of Psychiatry I Medical University of Innsbruck Innsbruck Austria; ^2^ Department of Psychiatry, Psychotherapy and Psychosomatics University Hospital of Psychiatry II Medical University of Innsbruck Innsbruck Austria; ^3^ Department of Psychiatry, Psychotherapy and Psychosomatics University Hospital of Medical Psychology Medical University of Innsbruck Innsbruck Austria Medical University of Innsbruck Innsbruck Austria; ^4^ Oncological Rehabilitation St. Veit im Pongau St. Veit im Pongau Austria

## Abstract

**Background:**

Implemenation of patient‐reported outcomes (PRO) like quality of life can add the patient's perspective to traditional clinical outcomes of cancer rehabilitation in a structured and standardized way.

**Aim:**

To present useful steps for a successful implementation of routine electronic patient‐reported outcomes (ePRO) monitoring. The presented steps are exemplified by describing the procedure applied in an Austrian inpatient cancer rehabilitation centre.

**Methods:**

The suggested implementation steps are presented based on the structure of the replicating effective programmes framework, which was used for developing a pragmatic implementation strategy.

**Results:**

We scheduled alternating trainings and process evaluations for audit and enhancement of procedures. In this way, the ePRO participation rates could be improved. Stakeholder involvement led to initiatives that included the integration of ePRO data into the medical discharge letter and the implementation of follow‐up assessments.

**Discussion:**

Tailored changes in assessment procedures enabled the successful implementation of ePRO, which has been shown to be feasible before and after cancer rehabilitation. The continuous involvement of stakeholders paved the way for further projects initiated by medical staff as users themselves (inclusion of PRO data in the discharge letter and a comprehensive ePRO follow‐up using a versatile online patient portal).

**What’s known**

- Patients are the experts for reporting their subjective health status and quality of life. The gold standard to assess such data is the use of validated patient‐reported outcome (PRO) questionnaires.
- Due to the improved detection and treatment of cancer, the number of people dealing with an oncological disease, surviving it and needing rehabilitation is increasing.

**What’s new**

- Implementing routine electronic quality of life assessments in oncology inpatient rehabilitation is feasible.
- An inclusive approach should involve all stakeholders to achieve the best result.
- Repeated evaluation and individual adjustments of the workflow support the adoption of new procedures.

## 1 BACKGROUND

### 1.1 Get involved in patient‐reported outcomes assessment

Because of increasing cancer incidence^1^ and improved survival rates,^2^ the number of patients living with cancer and needing high‐quality cancer rehabilitation will rise in the near future.

Increasingly, the patients’ perspective on their cancer disease is being assessed using patient‐reported outcomes (PRO), which are defined as patients’ self‐reports about their health status without being interpreted or altered by another person.^3^ They bring the focus to aspects of the patient's subjective experience that are related to his or her health status and treatment and that may be relevant for further medical interventions. PRO measures assess, eg, patients’ health‐related quality of life (QOL), disease‐ or treatment‐related symptoms, functioning and well‐being. Through PRO, the patient's subjective experience is quantified and can complement physician‐reported data in a standardised manner, enabling gaining a more accurate picture of the patient's health status^4^, ^5^, ^6^ and bringing up aspects (eg, sexual issues), which might otherwise go unnoticed or be underestimated by clinicians.^7^, ^8^, ^9^, ^10^, ^11^ Established PRO instruments have undergone an elaborate scientific background and solid psychometric testing and must be interpreted in terms of the meaning and clinical relevance of absolute scores or changes in scores in order to be medically instructive.

Over the whole course of cancer treatment, in which rehabilitation and follow‐up play an increasingly important role,^12^ PRO can be used as a standardised screening to promote efficient use of time in medical consultations, improve the communication between patients and their therapists, ensure treatment continuity and facilitate patient‐centred interventions as well as participatory decision‐making.^13^ Hence, similar to their benefits for routine active anti‐cancer care, PROs can also be used in rehabilitation to screen patients for special care needs, tailor interventions to screening results, evaluate the treatment progress and collect data for performance measurement, which can as well feed into Big Data analyses.^14^, ^15^ It is reported that especially vulnerable cancer survivors (older age, lower education, depressive symptoms) are at a higher risk of dropping out longitudinal follow‐up PRO assessments post‐active treatment.^16^ This might be associated with major problems in at least two respects: First, these patients might have less access to the care they actually need (especially if PRO data are used to support follow‐up care) and second, this creates a considerable positive bias in data possibly used for Big Data analyses. Despite the many similarities in the application of PRO in clinical routine and in oncological rehabilitation, special attention must be paid, for example, to ensure that the choice of assessment instruments and the timing of the assessments are appropriately adapted to the purpose and setting of data collection.

Traditionally, paper‐pencil questionnaires are used for PRO data collection; this is generally associated with relatively laborious working procedures (especially regarding data processing and storage), prone to data input errors and requiring vast human resources. Electronic PRO (ePRO) monitoring can collect data in a cost‐effective and secure way and, depending on the tolerance of missing values within the software, data quality is generally higher than that of traditional paper‐pencil assessments.^17^, ^18^ The data is immediately available digitally, automatically scored and graphically presented and may be linked to the patients’ electronic health record.^19^ Electronic data processing, therefore, allows the use of ePRO data straight after questionnaire completion during the medical encounter. ePRO monitoring and specialised assessment software are increasingly incorporated into oncology routine, most frequently in settings including patients before treatment or undergoing active treatment,^20^, ^21^, ^22^, ^23^ but targeting cancer survivors after treatment as well.^24^ With the intention to help overcoming common obstacles to implementing routine (e)PRO monitoring, consulting resources provide useful information and generic guidance.^25^, ^26^ To the best of our knowledge, the literature on the use of ePRO monitoring in cancer rehabilitation is, however, still scarce, especially on the standardisation of implementation procedures.

**FIGURE 1:**
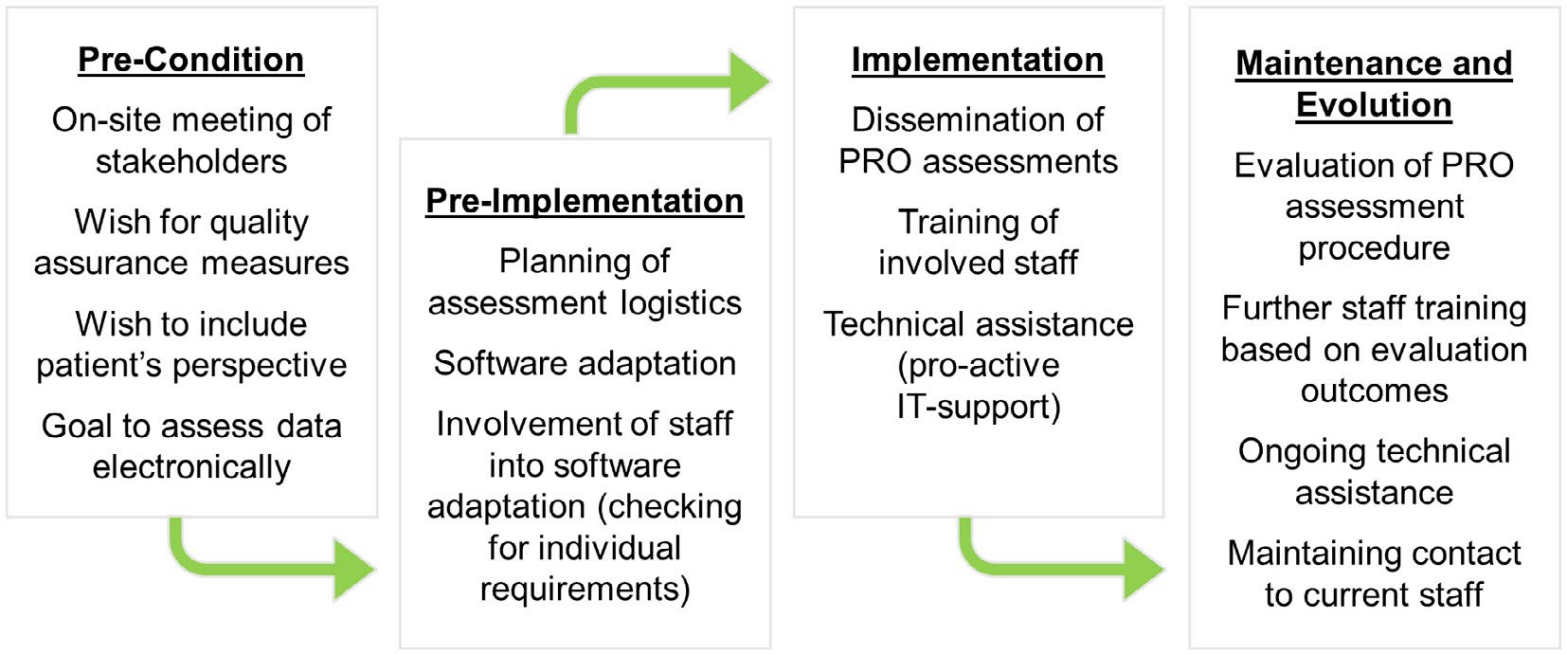
Components of the replicating effective programmes (REP) framework including actions taken for implementing ePRO

This paper presents useful steps for a successful implementation of routine ePRO monitoring in an oncological rehabilitation centre and explains by way of example how these steps were carried out in this particular case. The structure of the paper follows the four phases of the replicating effective programmes (REP)^27^ (see Figures 1 and 3).

## 2 PRE‐CONDITION

The oncology rehabilitation centre was opened in January 2014, provides 120 inpatient beds and offers a two to three‐week multidisciplinary inpatient rehabilitation programme for adult cancer patients (including various diagnoses) with a Karnofskyx index of >70.

### 2.1 Think about stakeholders and an implementation strategy

The medical director of the rehabilitation centre approached the software distributors to obtain their expertise regarding the following topics: developing an ePRO implementation strategy, providing the necessary software (including maintenance and IT support), data management and analysis. Besides communications via phone and e‐mail, six months before the opening of the rehabilitation centre an on‐site meeting of stakeholders (medical director, institution representatives, directors of therapy, administration officers, front desk staff, psycho‐oncologists and the software distributors) was held in order to develop a pragmatic implementation strategy on the basis of REP.^27^ Figure 1 provides an overview of the single process steps for each of the four REP‐phases.

## 3 PRE‐IMPLEMENTATION

### 3.1 Set up general procedures of the ePRO monitoring

Patients’ PRO data were assessed twice: before admission to rehabilitation and up to three days before discharge during their psycho‐oncology closing meeting. Front desk staff responsible for ePRO monitoring was trained within another facility of the rehabilitation provider following their established working routine, which was adopted in exactly the same manner. For this reason, the same PRO instruments were used as in the other institution. Furthermore, to keep procedures same way, PRO‐assessment was initially implemented as paper‐pencil assessment (patients received PRO instruments by mail, including a return envelope) and changed to ePRO after a six‐month adaptation phase in June 2014 (patients received an information sheet providing a unique user name and initial password for online ePRO access). Irrespective of data collection mode, patients received a reminder phone call by the front desk staff, if they had not provided their data 10 days before admission.

### 3.2 Define outcome parameters for successful ePRO implementation

Evaluating procedures is key to in order to recognise whether they work under real conditions, as they should. It is, therefore, important to define parameters that indicate whether certain goals have been achieved. To evaluate the success of ePRO implementation, the following outcome parameters were determined by the on‐site stakeholders:

1. feasibility of ePRO assessments (ie, how practicable is ePRO in this given institution), operationalised by feedback prompted by users (medical and administrative personnel), feedback regarding the effect of applied adjustments of procedures;
2. patients’ acceptance (ie, do patients complete ePRO), operationalised by patients’ response rates (≥70%; in literature, patients’ response rates to mailed or electronically distributed PRO ranges between 31% and 65%^28^, ^29^, ^30^, ^31^) and unstructured collection of feedback prompted by patients;
3. professional users’ acceptance (ie, does the centre personnel, in particular medical and administrative personnel, engage in ePRO), operationalised by training participation rates (≥70%, corresponding to patients’ response rates), unstructured collection of professional users’ feedback.

### 3.3 Choose ePRO measures appropriate for your purpose

Completion of the PRO assessment required approximately 30 to 45 minutes and included the followings instruments:

- the European Organisation for Research and Treatment of Cancer Quality of Life Questionnaire Core 30 (EORTC QLQ‐C30)^32^ (cancer‐specific QOL questionnaire)
- the Hospital Anxiety and Depression Scale German Version (HADS‐D)^33^ (for assessment of anxiety and depression)
- the Short Screening Scale for DSM‐IV Post‐Traumatic Stress Disorder (SSS‐PSD)^34^ (assessing elevated arousal and symptoms of avoidance)
- the EuroQOL‐5‐Dimensions (EQ‐5D‐3L)^35^ (generic QOL instrument)
- four additional yes‐no questions assessing the patient's wish for a special psycho‐oncology treatment focus/type of treatment, previous diagnosis of any psychiatric disorder and previous psychotherapeutic treatment

### 3.4 Define how ePRO data feed into routine care

ePRO scores guided the frequency and focus of psycho‐oncology sessions, using patients’ HADS anxiety or depression scale scores and their special interest in psycho‐oncology treatment. If no (e)PRO data were available, the default frequency of sessions was assigned.

### 3.5 Choose a software for ePRO monitoring

CHES is a software especially developed for electronic collection of PRO data (eg, in daily clinical practice, study monitoring, online patient portals, clinical data bases and registries) and its calculation, presentation and secure storage.^36^ Based on the globally accepted Health Level 7 (HL7) standard, an interface enabled interoperability with other clinical information systems, easing patient administration. Each patient registered for rehabilitation automatically received a CHES account and personal log‐in credentials for online ePRO completion. As patients keep their accounts beyond their rehabilitation stay, follow‐up assessments are possible.

**FIGURE 2:**
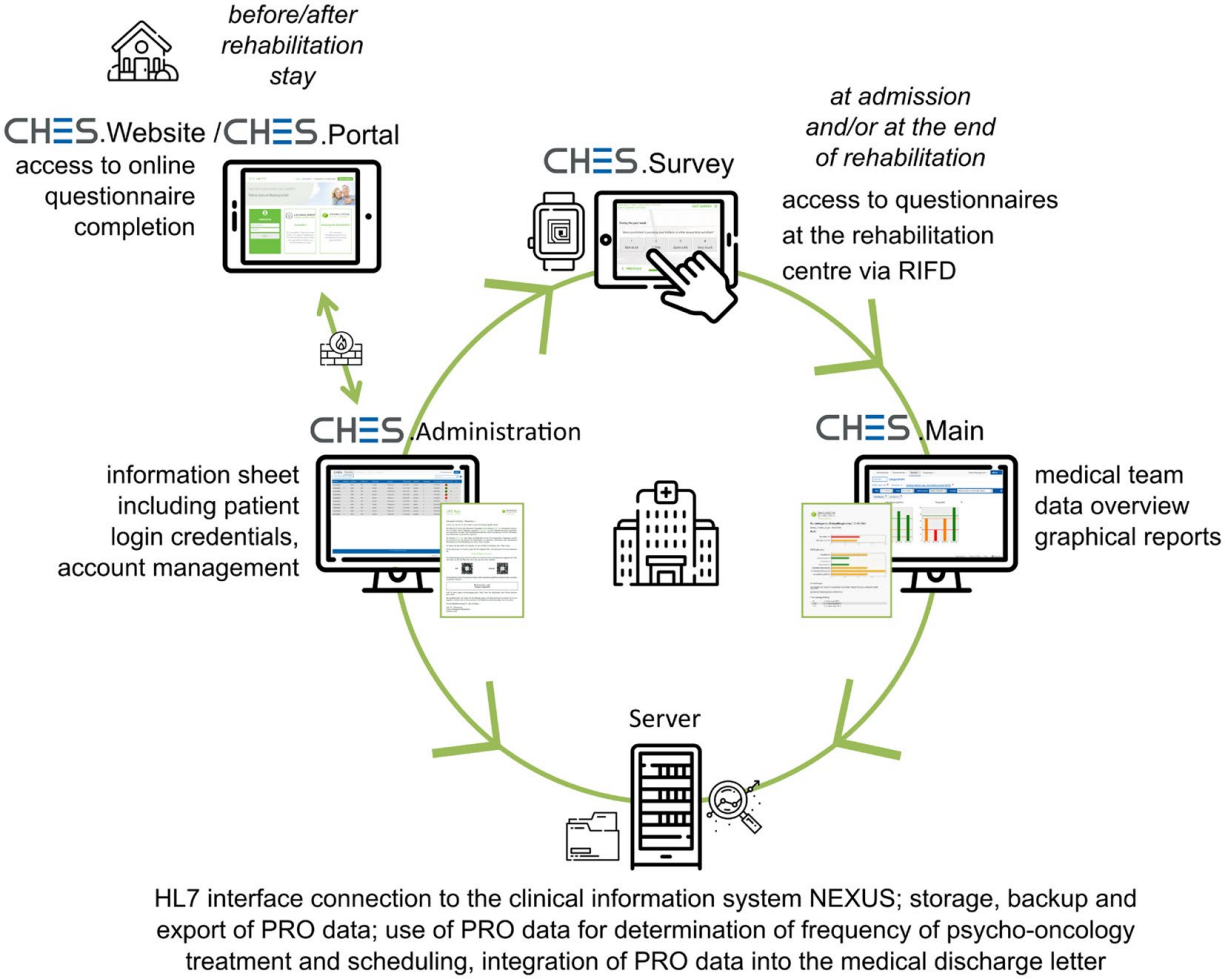
Schematic diagram of the software functionalities used at the rehabilitation centre for ePRO assessment

Allocated user rights regulate access to different software features and differentiate between medical staff, administration officers and patients. Next to administrative features, ePRO data can be remotely collected via an online patient website/portal or onsite using a survey application. Health care professionals can access patients’ data, generate cross‐sectional or longitudinal reports, add ePRO data to the patient's hospital discharge letter and enter/retrieve information about specific interventions. Figure 2 provides a schematic diagram of the software functionalities.

## 4 IMPLEMENTATION

**FIGURE 3:**
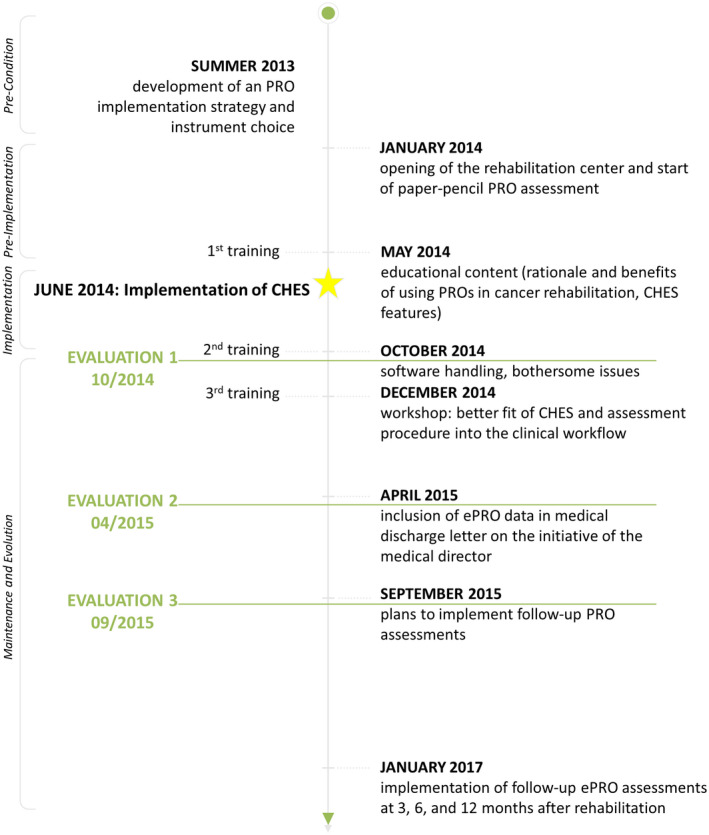
Flow‐chart of (e)PRO implementation at the rehabilitation centre

The implementation and evaluation process consisted of each three partially overlapping user training sessions and evaluations. Please refer to Figure 3 for a flow chart of the (e)PRO and CHES implementation process.

### 4.1 Check whether ePRO feeds into routine care as intended

Based on ePRO‐scores, the default frequency of four one‐on‐one psycho‐oncology sessions was increased to up to eight sessions. Neither administrative staff nor psycho‐oncologists reported any problems using the recommendations based on the automated algorithm or individually adjusted treatment plans to be inadequate.

### 4.2 Provide user‐centred trainings

Members of the software company conducted three training sessions for rehabilitation centre personnel involved in ePRO, addressing different aspects of PRO assessments and encouraging open discussion:

1. May 2014: Educational session regarding the rationale for using PRO in cancer rehabilitation, benefits for patients and health care professionals and presentation of software features.
2. October 2014: On‐site troubleshooting analysing data collection procedure and solving technical issues.
3. December 2014: Group discussion on how ePRO could be more efficiently embedded into the clinical workflow.

Approached staff members and stakeholders showed great interest in the provided trainings and evaluations with very high participation rates (at least 80%, only very few nonparticipants resulting from illness or vacation), even though attendance was voluntary.

## 5 MAINTENANCE AND EVOLUTION

### 5.1 Evaluate whether everything works as planned

During a two‐day participating observation of a software company member in October 2014, practical help in software handling was provided. The staff's feedback showed that the response rates were rather low (22%) and that they did not feel well prepared for ePRO‐related critical inquiries of patients. To improve response rates, the cover letter to patients was refined, presenting ePRO as a substantial part of the routine rehabilitation procedure. Furthermore, phone support for front desk stuff was provided between October 2014 and December 2014. By the end of December 2014, patients’ PRO response rates had increased from 22% to 83%.

Many patients gave a negative feedback about the questionnaire SSS‐PSD being intrusive and not appropriate. As the adequacy of this measure with regard to content is disputable in a rehabilitation context, it was excluded from the ePRO assessment.

### 5.2 Improve and re‐evaluate procedures accordingly

In April 2015, a stakeholder (medical director, institution representatives, directors of therapy, administration officers, front desk staff, psycho‐oncologists and the software distributors) update meeting took place, discussing the current status of the ePRO assessment procedure. Overall, no profound problems, regarding neither IT nor patient‐related aspects, had arisen since the first evaluation.

Response rates of patients in ePRO assessments at the beginning of rehabilitation could be further increased to 98%. Since then, participation rates have settled at around 90%.

Front desk staff reported reminder phone calls (if patients did not provide their PRO data 10 days before admission) to be less aversive for them, as the provided phone support made them feel better prepared for questions raised by patients. After the deletion of the SSS‐PSD questionnaire, negative feedback from participating patients considerably decreased.

On the initiative of the medical director, it was decided to include selected ePRO scores into the medical discharge letter. The scores of six scales of the EORTC QLQ‐C30 (Physical Functioning, Emotional Functioning, Cognitive Functioning, Fatigue, Pain and Sleeping Disturbances) were added by providing a table and bar charts. In order to facilitate the interpretation of the ePRO data for, eg, general practitioners, who are typically important medical contact persons for patients and usually not very familiar with ePRO data, norm values were also included.

### 5.3 Repeat as often as necessary

In September 2015, a further stakeholder meeting was dedicated to the discussion of how ePRO assessments can be successfully extended into clinical routine, providing patients’ self‐report data to all health care professionals for their individual use during patient encounter. In addition, the meeting included in‐depth considerations about a feasible ePRO follow‐up procedure.

### 5.4 Check whether your outcome parameters for successful ePRO‐implementation have been met

The predefined outcome parameters for successful implementation support the feasibility and acceptability of routine ePRO assessments:

1. Feasibility: Specific interventions led to improved user feedback: case‐specific software training, advanced information of users about the rationale and benefits of ePRO, discussion about changes in procedures for less disruption of the clinical workflow and adjustments of the clinical information system.
2. Patients’ response rates: After the second training and the adaptation of the cover letter, patients’ initial participation rates have been consistently high (increase from 22% to 83% and later 98%) and patients’ negative feedback has dropped to almost zero after adjustment of the used ePRO instruments (deletion of the SSS‐PSD).
3. Professional users’ acceptance: Each training was attended by at least 80% of professional users, who gave predominantly positive feedback after evaluations. They were open to other areas of application of ePRO and engaged themselves in initiatives for further use of ePRO data.

### 5.5 Think ahead

The existing IT infrastructure of the rehabilitation centre for the collection and use of ePRO enables the conduct of, eg, follow‐up studies. A 1‐year follow‐up procedure (with assessments at 3, 6 and 12‐month post‐rehabilitation) was implemented in early January 2017 and will contribute to the long‐term investigation of rehabilitation effects, which play an important role in terms of rehabilitation services’ sustainability (eg, long‐term benefit for patients, cost‐effectiveness of rehabilitation). Reluctance to conduct PRO assessments continuously often relies on the fact that patients do not see any personal benefit in them and they get tired of just filling in questionnaires. Having access to their personal PRO scores and to tailored self‐help advice, however, involves the patients in their care, provides feedback and can thus generate individual benefit and a greater willingness to participate. This very strategy is used to increase the attractiveness of follow‐up ePRO assessments to patients and to prevent a common decrease in response rates during follow‐up assessments after initially high inclusion rates.^37^ The ongoing collection of follow‐up data will accumulate a large pool of PRO data, which might be of interest to data mining or analytical reporting in the future.

## 6 DISCUSSION

We presented useful steps for achieving a successful implementation of a routine ePRO assessment and exemplified them by describing the process of such an implementation at an oncological inpatient rehabilitation centre. Data on patients’ QOL and psychological distress before and after rehabilitation are reported elsewhere.^38^, ^39^

As suggested by Snyder et al,^26^ the time structure of oncology rehabilitation offers practicable possibilities to smoothly incorporate ePRO in the given schedule. The fact that the assessment of ePRO has been included in the workflow of the rehabilitation centre from the very start has avoided any changes or disruptions as a result of (e)PRO assessment. Web‐based ePRO assessments showed to be a feasible method for electronic data collection in cancer rehabilitation patients, which might be even preferred by patients and provide better data quality than paper‐pencil assessments.^37^, ^40^

### 6.1 Learn your lessons

Though already putting a lot of effort into the pre‐condition and pre‐implementation phases, we can report on some important lessons learned within the presented implementation process. The importance of the careful choice of appropriate ePRO instruments^41^ was underlined by the fact that many patients reported to feel irritated by the SSS‐PDF, a questionnaire focusing on posttraumatic stress disorder. Furthermore, especially patients’ needs seemed to have been not adequately met in the set‐up of ePRO presentation and collection. The strong focus on the wishes and needs of professional users concerning IT‐related issues and on the provision of an easy functioning and reliable software system may have distracted the attention from aspects regarding user information and patients’ motivation to engage in ePRO assessments. Other studies show that especially patients’ positive attitudes towards PRO are linked to a clearly perceivable personal benefit of providing this kind of data.^42^, ^43^ The initially low response rates suggested that many patients did not recognise any individual gain in (e)PRO assessments and seemed to perceive it as unnecessary add‐on to regular rehabilitation procedures. Performing a detailed stakeholder analysis would have prevented the one‐sided emphasis on the centre personnel by including patients and patient representatives on the stakeholder list. This could have prevented the aforementioned shortcoming by instantly developing a more adequate patient information sheet.

**FIGURE 4:**
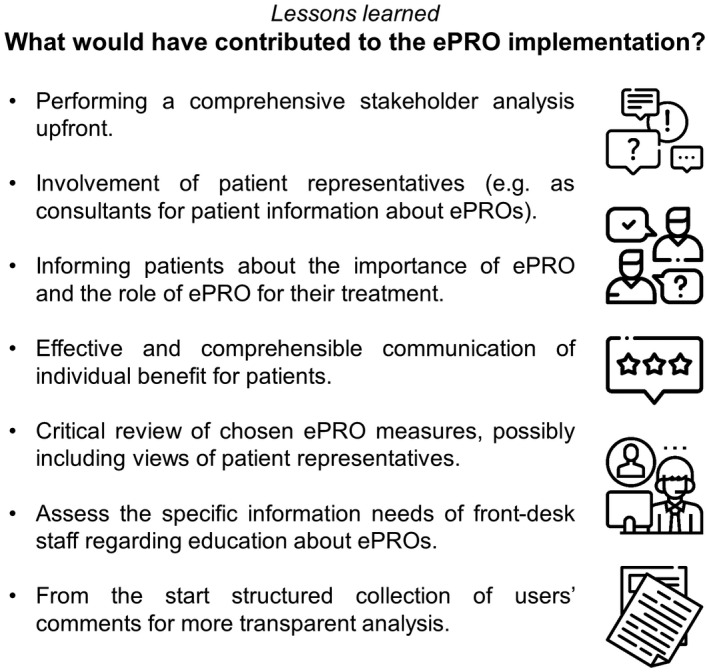
Lessons learned: what would have contributed to the ePRO implementation?

The second evaluation made obvious that the attitude of those staff members who had contact with patients posing critical questions about ePRO assessments plays an important role in their interaction with inquiring patients.^44^ It is possible that the initial training before ePRO implementation lacked sufficient institution‐specific educational content, as one major criticism of front desk staff was that their unfamiliarity with the concept, use and benefits of PRO as well as IT‐related questions often made them feel uncomfortable in ePRO‐related patient contact. An in‐depth stakeholder analysis could also have been helpful in this respect by identifying the specific information needs at an early stage. The approach to combine several steps of ePRO assessment procedure evaluation with concerted training appears to have been a good way to enhance user involvement, which in turn seems to have positively influenced the interaction with patients, the collection of ePRO data and response rates. These observations go in line with other studies that report well informed, supportive staff and motivated patients (in a sense of perceiving the completion of ePRO to be relevant both for them personally as well as for their health care providers, eg, by triggering interventions) to be beneficial for successful ePRO assessments.^45^, ^46^, ^47^ A summary of these most important lessons learned is presented in Figure 4 and can serve as a checklist of easily avoidable pitfalls.

### 6.2 Conclusion

The implementation of ePRO prior to and after inpatient cancer rehabilitation was successful and feasible. This was achieved by following a pragmatic implementation strategy, adjusting procedures according to user feedback, conducting on‐site evaluations and providing tailored user training. Different stakeholders were involved over the whole course of implementation. Based on this inclusive approach, stakeholders engaged in further projects setting up follow‐up monitoring and integrating PRO data in the medical discharge letter. Strategic implementation and ongoing evaluation appear to ease the adoption of new techniques.
